# Supplementary material for: Mitochondrial damage drives T-cell immunometabolic paralysis after major surgery
Source: EMBO Mol Med. 2025 Nov 3;17(12):3329–54. doi: 10.1038/s44321-025-00324-1 (PMC12686421; doi:10.1038/s44321-025-00324-1)
Supplement: Supplementary file 1 — Table EV1 [file 44321_2025_324_MOESM1_ESM.pdf]

Table EV1: qRT-PCR Primers Probes and Assays

Target	Primer for	Primer rev	Probe
TBP	5’ GAACATCATGGATCAGAACAACA 3’	5’ ATAGGGATTCCGGGAGTCAT 3’	87
RPL13A	5’ CTG GAC CGT CTC AAG GTG TT 3’	5’ GCC CCA GAT AGG CAA ACT T 3’	74
PRF1	5’ CACTCACAGGCAGCCAACT 3’	5’ GGGAGTGTGTACCACATGGA 3’	26
GZMB	5’ GGGGGACCCAGAGATTAAAA 3’	5’ CCATTGTTTCGTCCATAGGAG 3’	37
IFNy	5’ GGCATTTTGAAGAATTGGAAAG 3’	5’ TTTGGATGCTCTGGTCATCTT 3’	21
NOX2	5‘GGC TTC CTC AGC TAC AAC ATC T 3’	5’ GTG CAC AGC AAA GTG ATT GG 3’	20
MPO	5’ AAG CTC CGG GAT GGT GAT 3’	5’ GAT CCG GGG CAA TGA GAT 3’	81
**Multiplexing Assay BioRad #**			
TBP	FAM 12001950 – Cy5 10031231		
RPL13A	FAM 12001950 – HEX 10031228		
GZMB	HEX 10031228		
IFNy	FAM 12001950 – Cy510031231		
PRF1	HEX 1003122		
