# Supplementary figures and images for: Mitochondrial damage drives T-cell immunometabolic paralysis after major surgery
Source: EMBO Mol Med. 2025 Nov 3;17(12):3329–54. doi: 10.1038/s44321-025-00324-1 (PMC12686421; doi:10.1038/s44321-025-00324-1)

Figure 4 F

OXPPOS Complexes

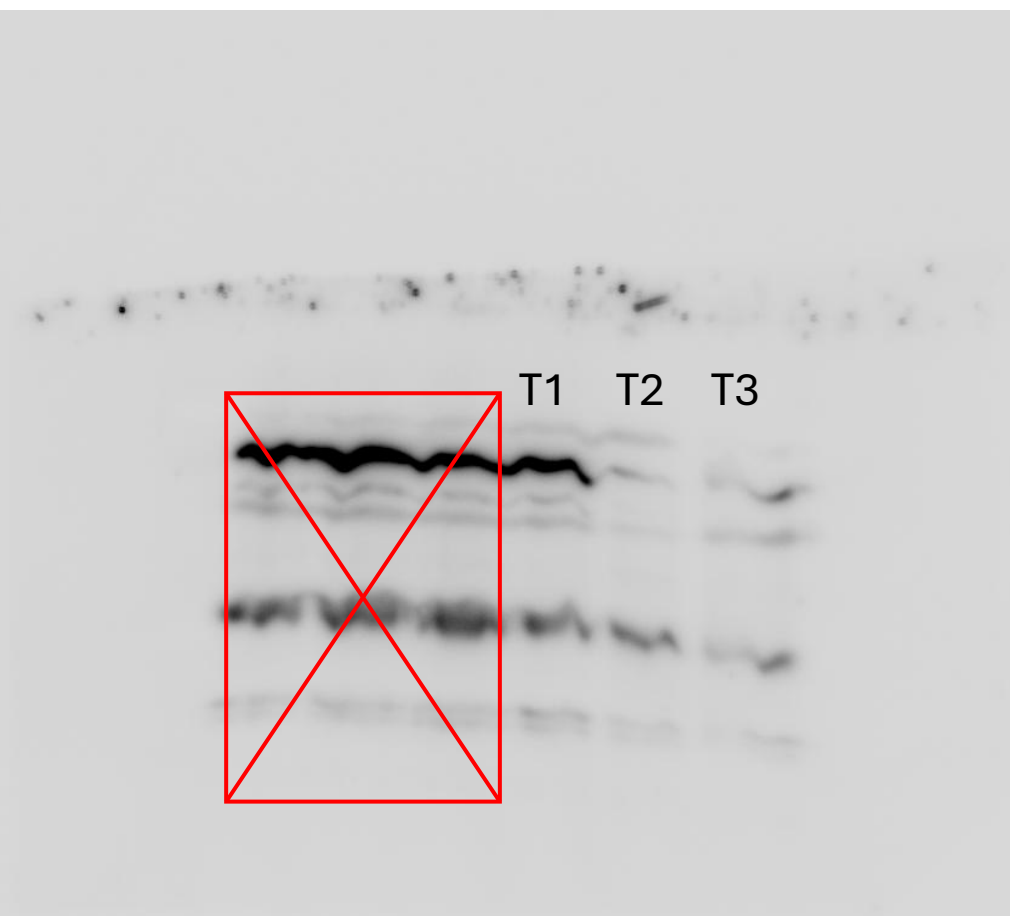

Beta Actin

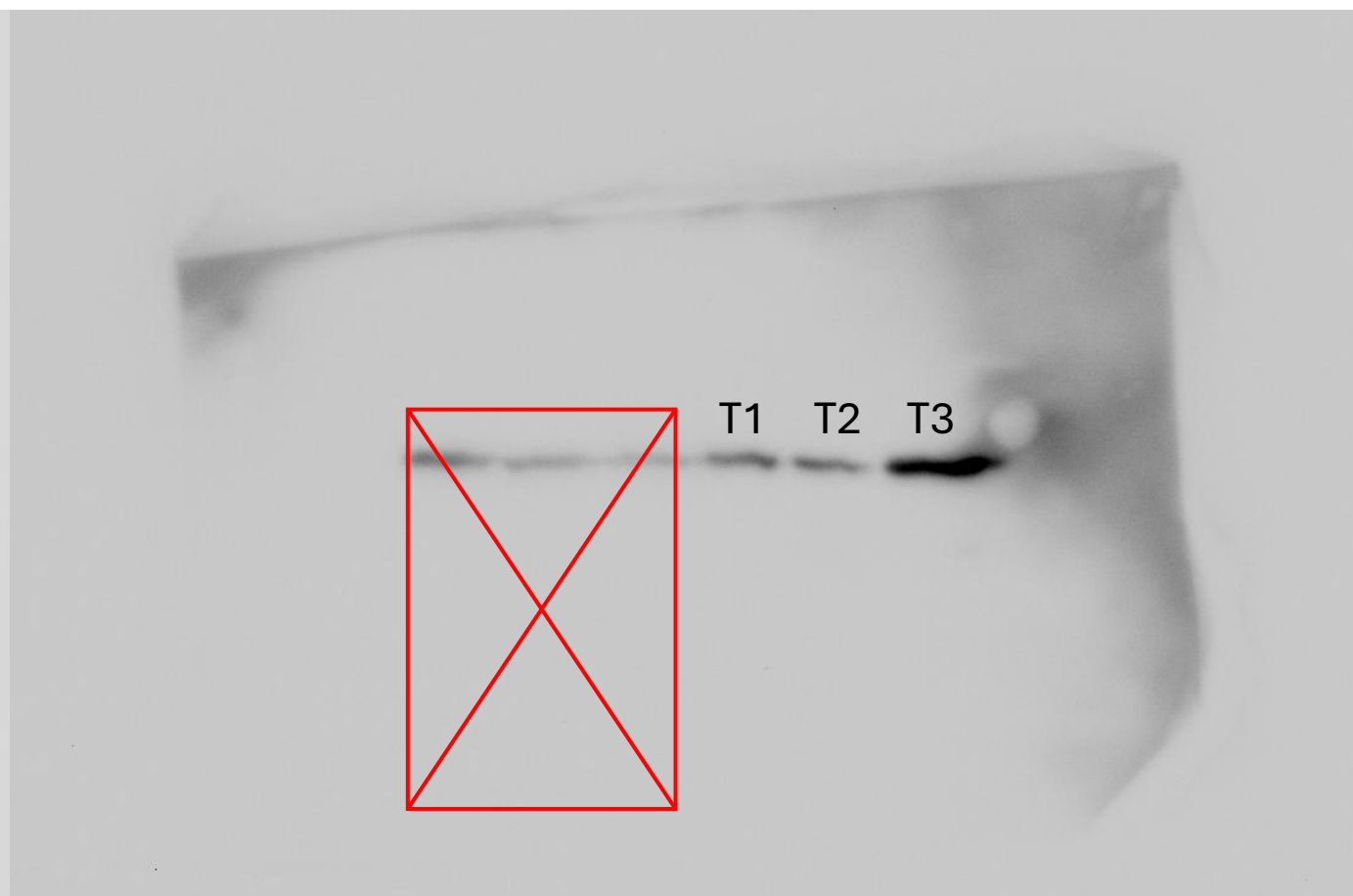

Supplement: Supplementary file 6 — Source data Fig. 4 [file 44321_2025_324_MOESM6_ESM.zip › Fig 4F.pdf]

Figure 5D

OPA1

MFN2

HSP60

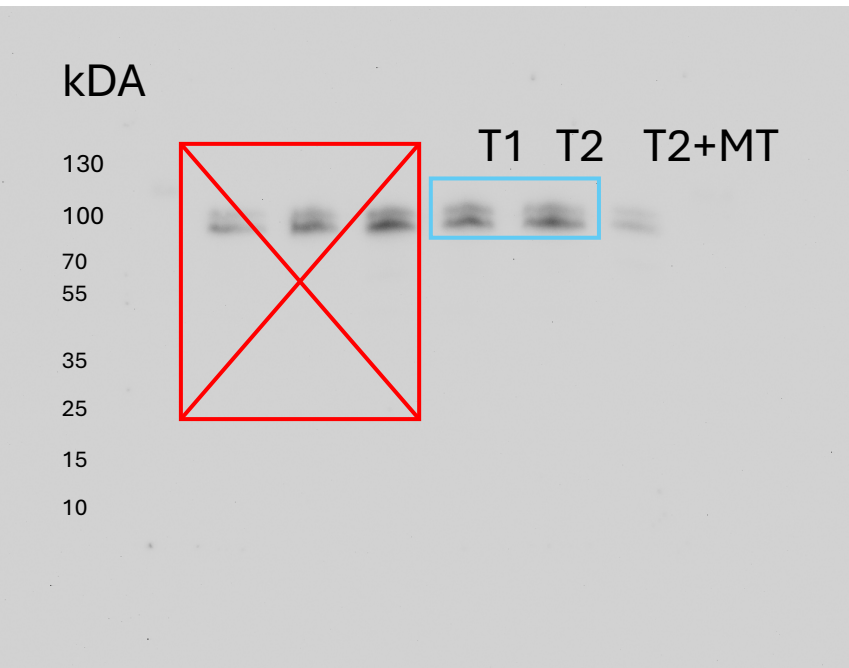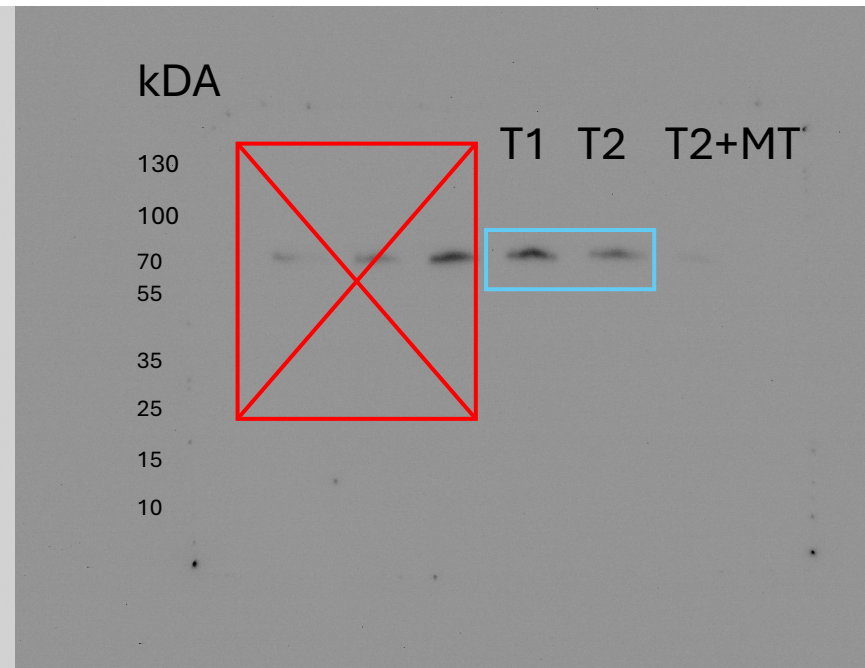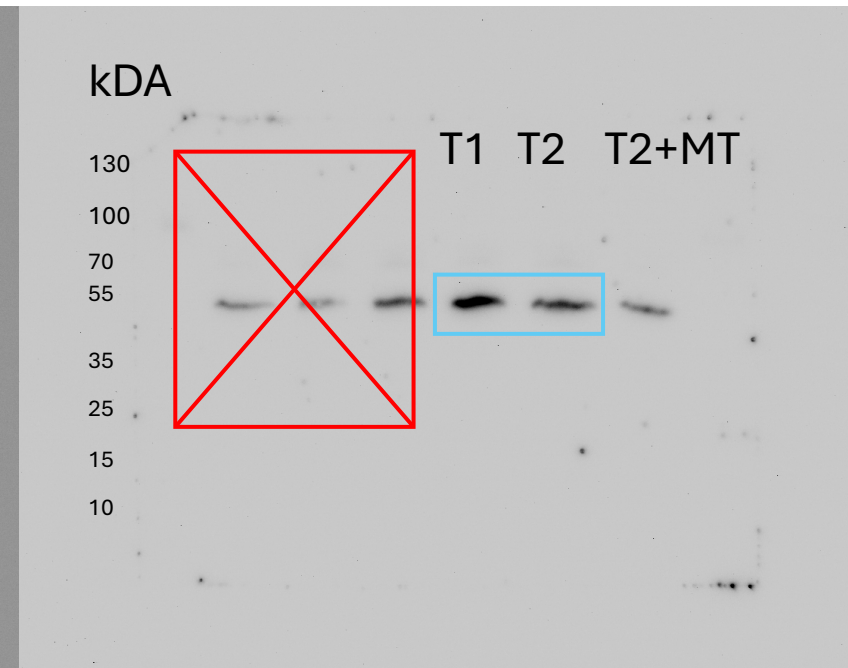

Figure 5D

DRP1

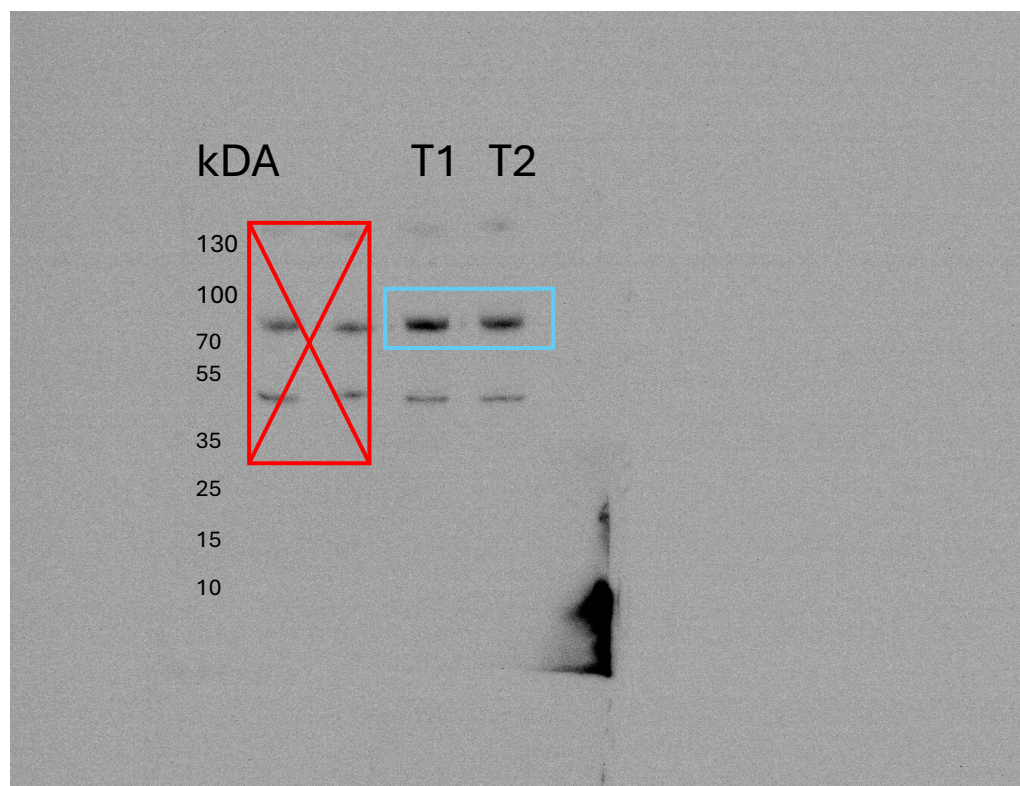

HSP60

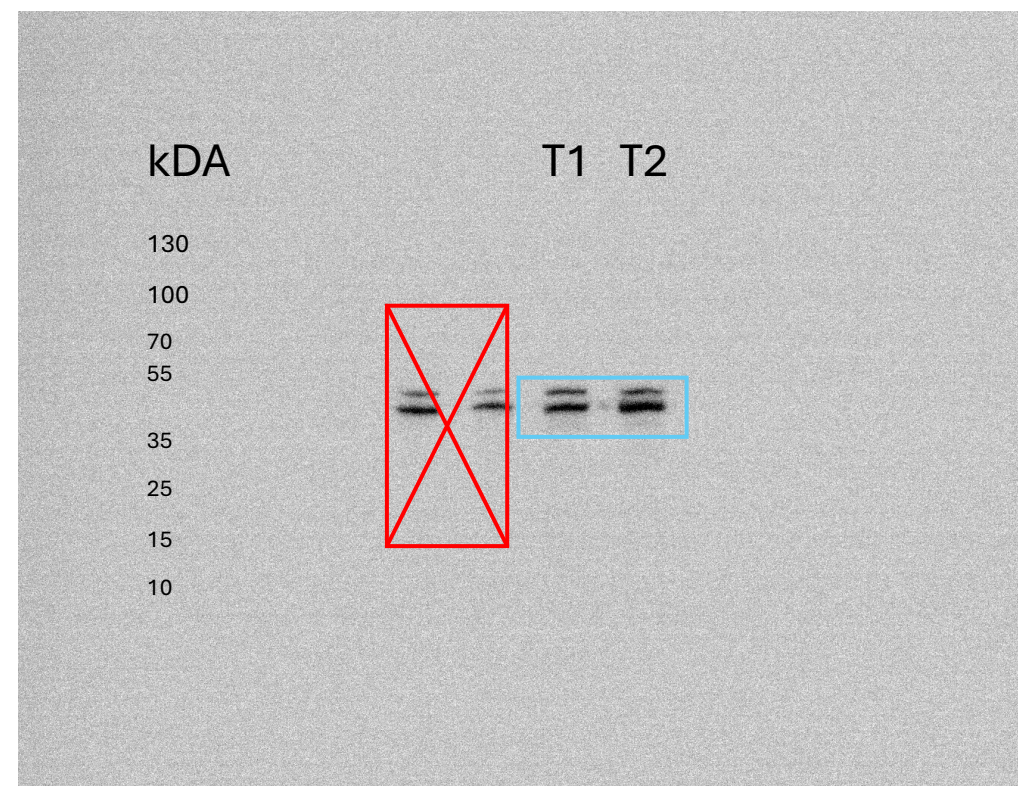

Supplement: Supplementary file 7 — Source data Fig. 5 [file 44321_2025_324_MOESM7_ESM.zip › Fig 5D.pdf]

Figure 5C

OXPHOS Complexes

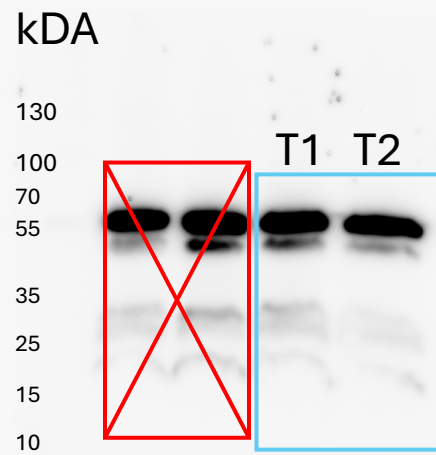

HSP60

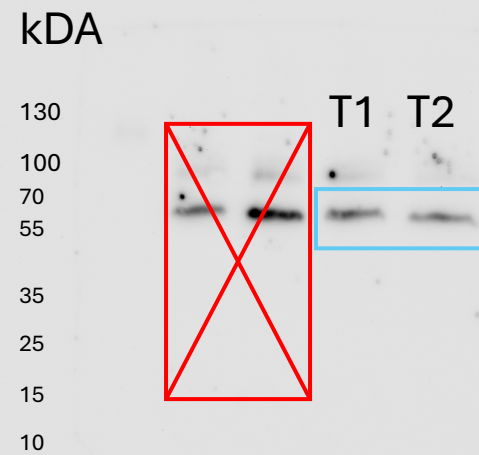

Supplement: Supplementary file 7 — Source data Fig. 5 [file 44321_2025_324_MOESM7_ESM.zip › Fig 5C.pdf]

Figure 6F

DRP1

OPA1

HSP60

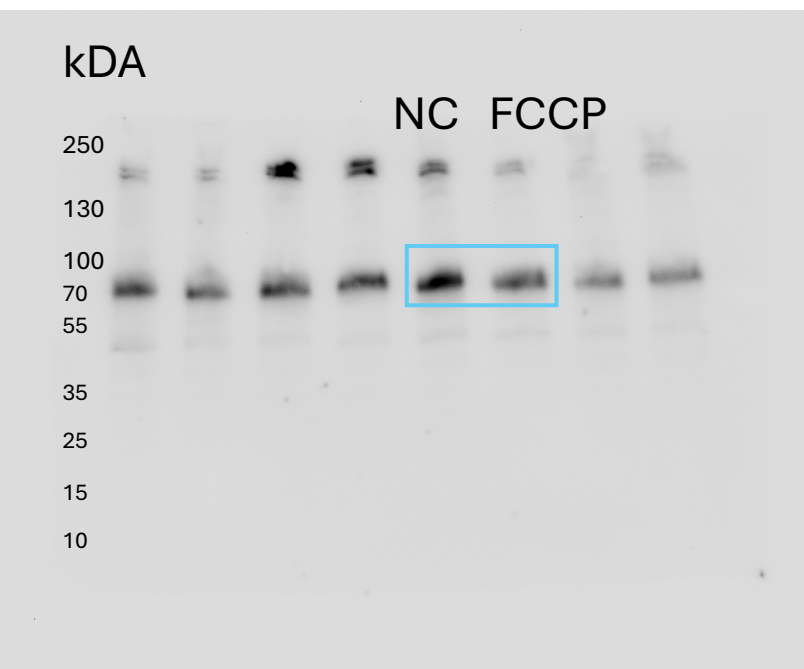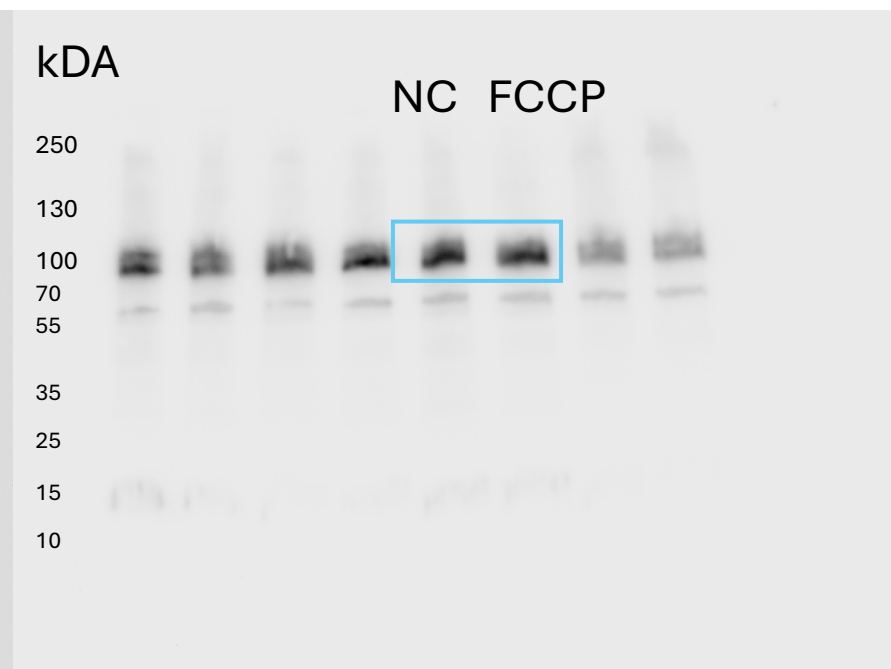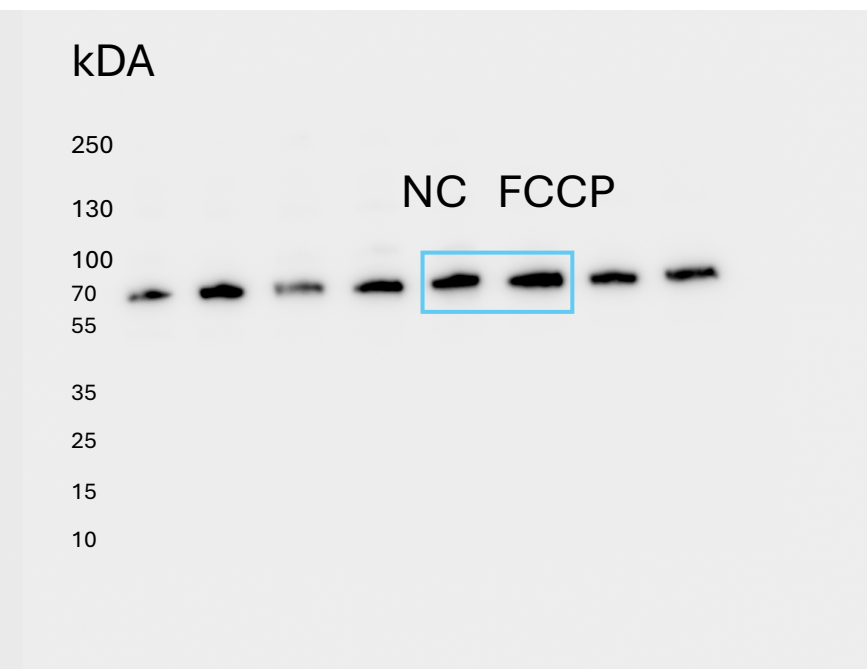

Figure 6F

MFN2

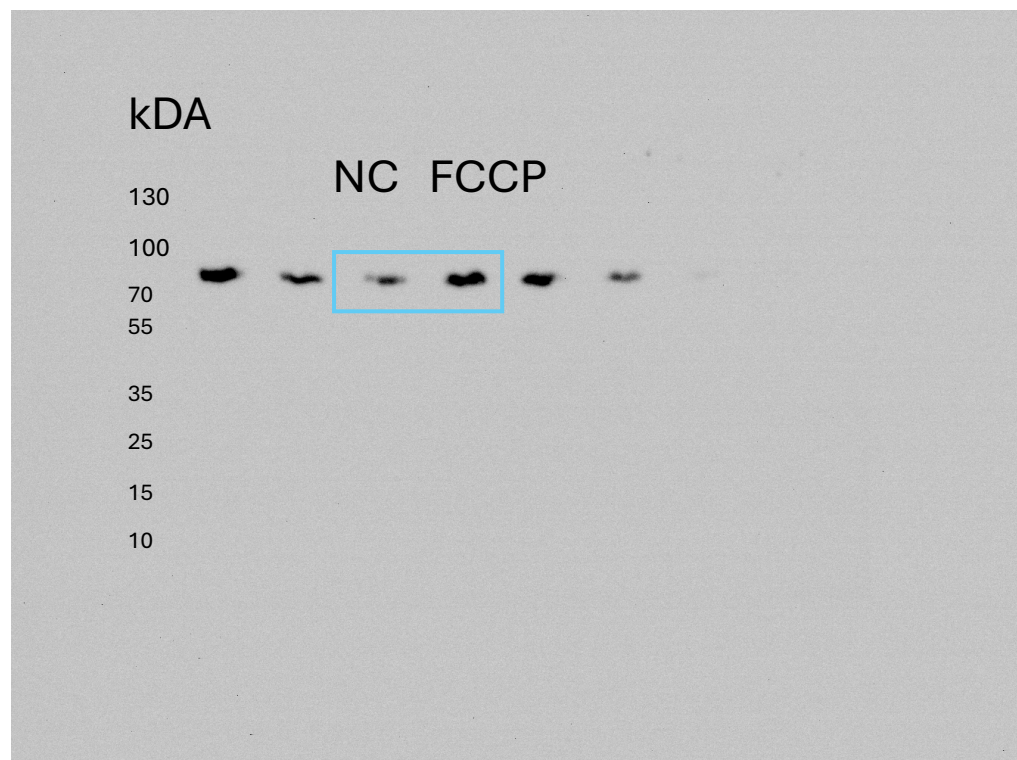

HSP60

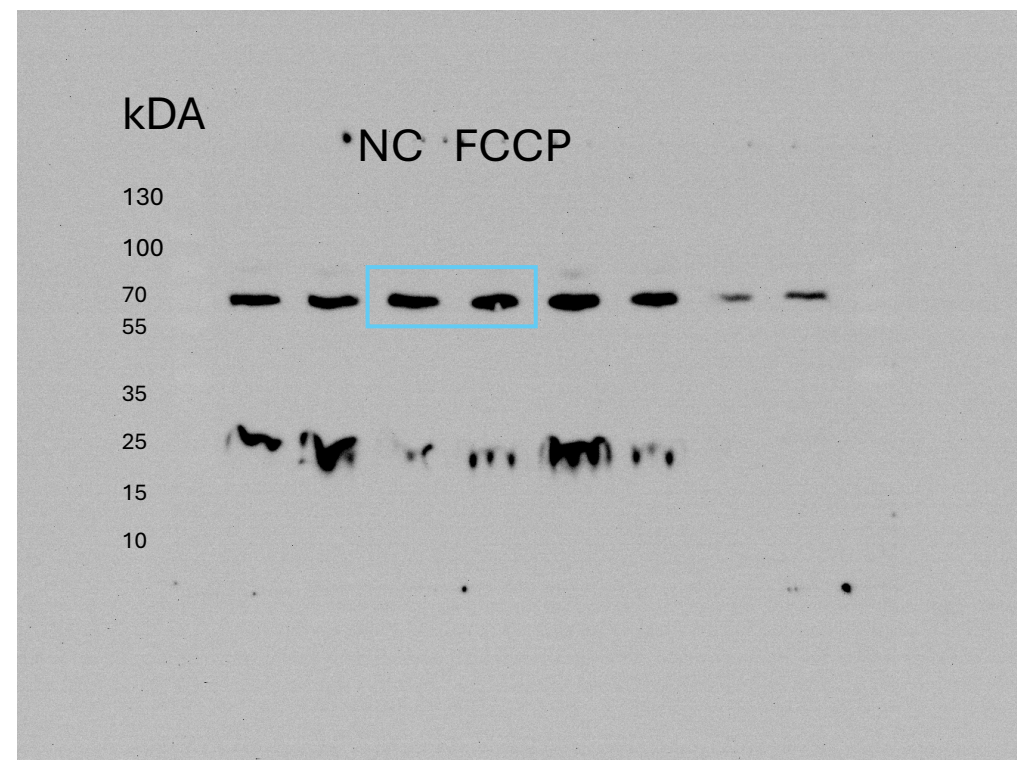

Supplement: Supplementary file 8 — Source data Fig. 6 [file 44321_2025_324_MOESM8_ESM.zip › Fig 6F.pdf]
