# Supplementary material for: Mitochondrial damage drives T-cell immunometabolic paralysis after major surgery
Source: EMBO Mol Med. 2025 Nov 3;17(12):3329–54. doi: 10.1038/s44321-025-00324-1 (PMC12686421; doi:10.1038/s44321-025-00324-1)
Supplement: Supplementary file 10 — Expanded View Figures [file 44321_2025_324_MOESM10_ESM.pdf]

Expanded View Figures

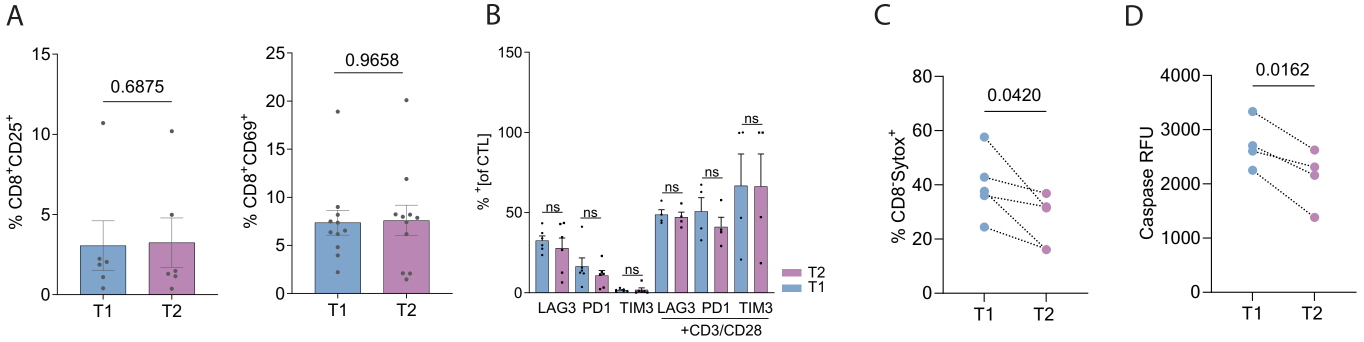

Figure EV1. Post surgical immunosuppression (PITC) of CTL.

(**A**) Flow cytometric analysis (FCA) of CD25 (left) and CD69 (right) staining and quantification in CTL, $n = 6/11$ individual patients. (**B**) FCA of lymphocyte-activation gene 3 (LAG3), programmed cell death protein 1 (PD1), and T-cell immunoglobulin and mucin-domain containing-3 (TIM3) staining and quantification as indicated, $n = 6/4$ (not activated/+CD3/CD28) individual patients. *P* values (left to right): 0.2842, 0.2188, 0.6875, 0.5494, 0.1250, and >0.9999. (**C**) Quantification of relative cytotoxicity using a flow cytometric killing assay, $n = 5$ individual patients. (**D**) Quantification of caspase-3 activity reporter using a CTL-K562 killing assay, $n = 4$ individual patients. Data were represented as mean ± SEM with dots indicating individual values (**A**, **B**) or as individual data points (**C**, **D**). *P* values: as indicated, paired *t*-test or Wilcoxon matched-pairs signed-rank test, as appropriate.

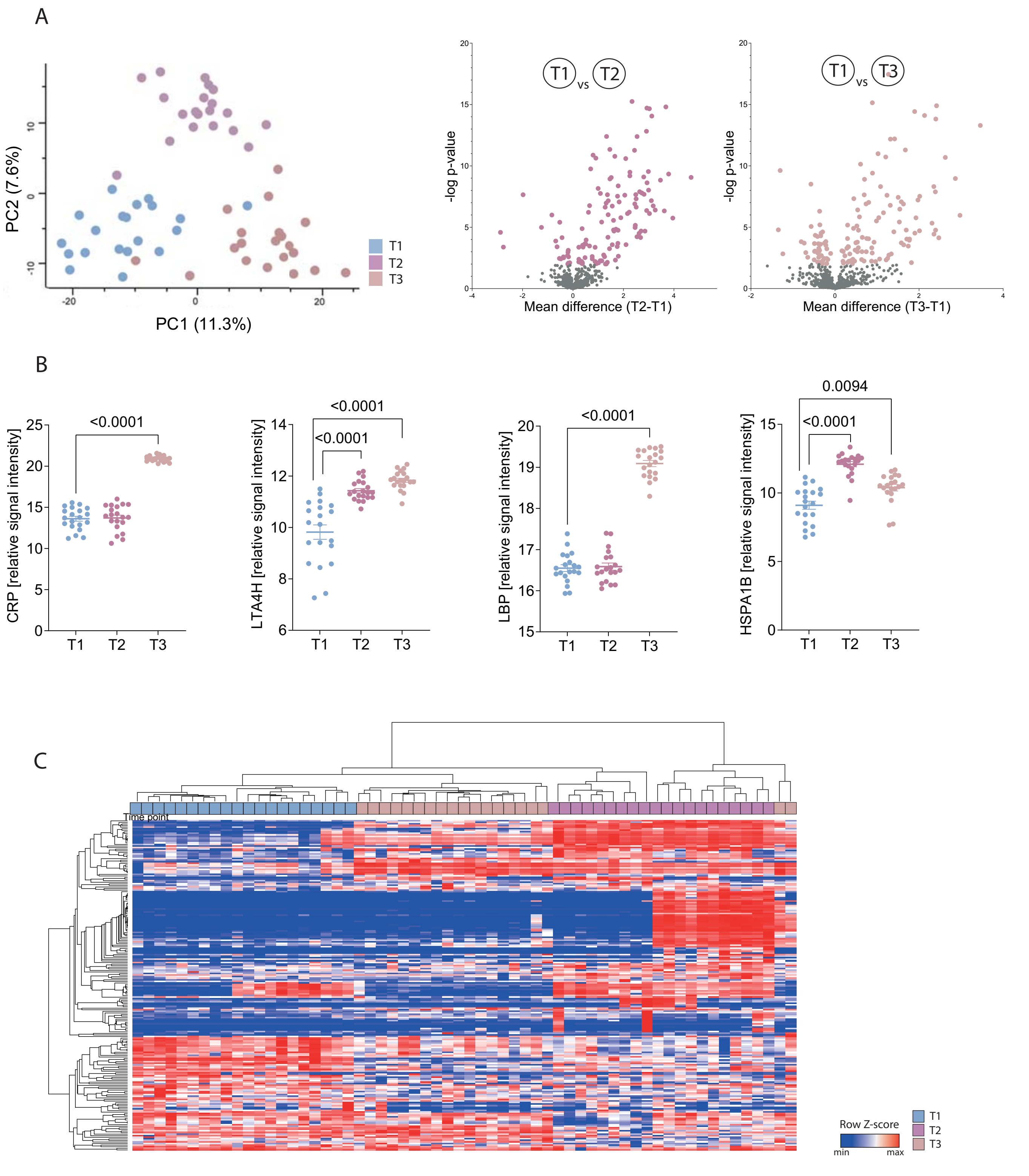

Figure EV2. Serum proteomics and metabolomics reveal an oxidative stress signature after major surgery.

(**A**) Principal component analysis depicting proteomic profiling results during the course of major surgery, time points as indicated (left) and Volcano plots for protein abundance of comparisons as noted on the top. Proteins with −log *p* value >2 are colored, $n = 20$ individual patients. (**B**) Relative signal intensity of serum proteins during the course of major surgery as indicated, $n = 20$ individual patients. Mean ± SEM with dots representing individual values, *p* values as indicated, paired *t*-test. (**C**) Heat map depicting quantity of significantly differentially expressed serum metabolites. Raw z-scores and time points as indicated, $n = 20$ individual patients.

 © The Author(s)

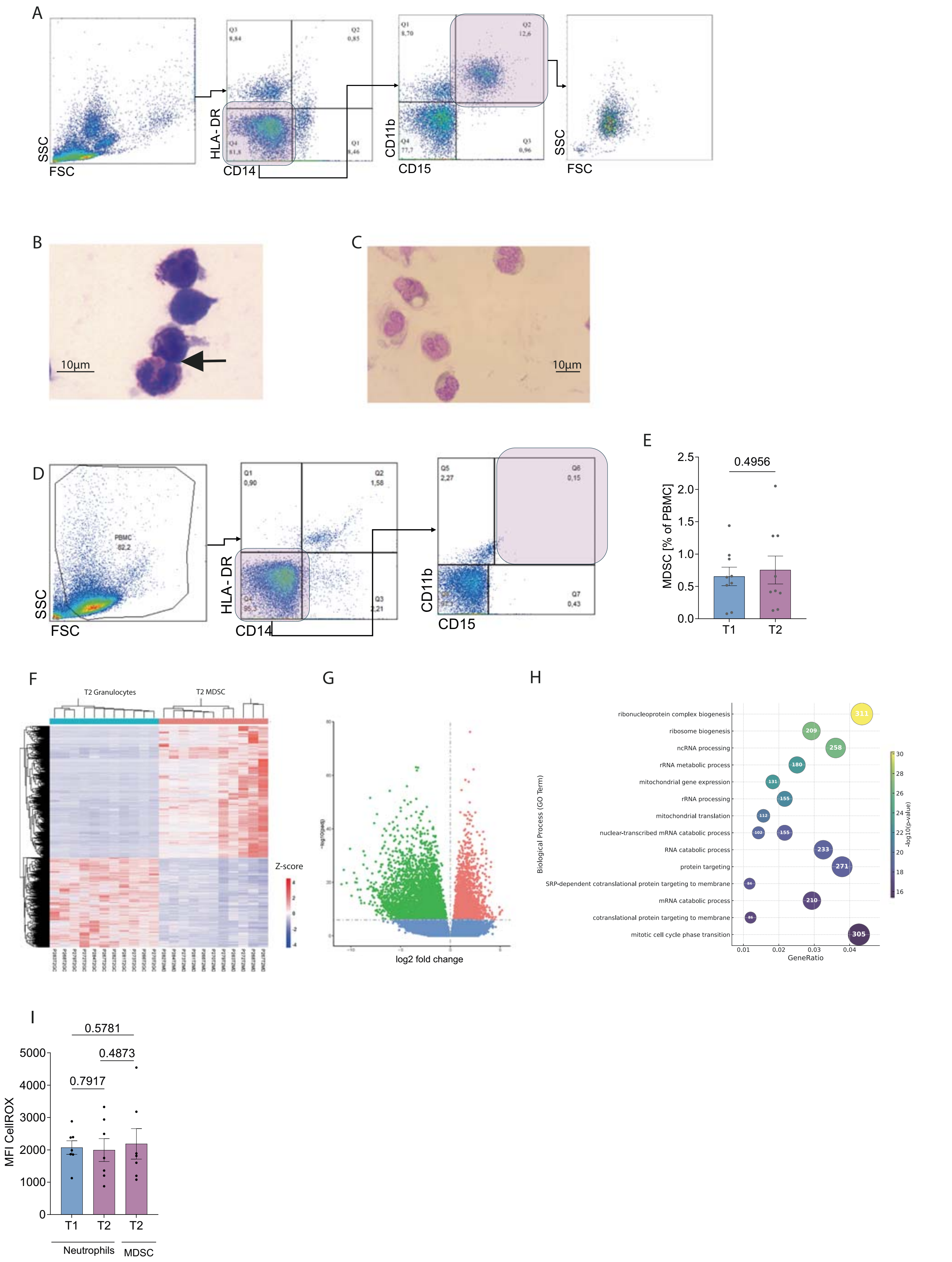
A
SSC
FSC
HLA-DR
CD14
CD11b
CD15
B
10µm
C
10µm
D
PBMC
82,2
E
0.4956
MDSC [% of PBMC]
T1
T2
F
T2 Granulocytes
T2 MDSC
Z-score
G
log2 fold change
H
ribonucleoprotein complex biogenesis
ribosome biogenesis
ncRNA processing
rRNA metabolic process
mitochondrial gene expression
rRNA processing
mitochondrial translation
nuclear-transcribed mRNA catabolic process
RNA catabolic process
protein targeting
SRP-dependent cotranslational protein targeting to membrane
mRNA catabolic process
cotranslational protein targeting to membrane
mitotic cell cycle phase transition
Biological Process (GO Term)
GeneRatio
-log10(p-value)
I
0.5781
0.4873
0.7917
MFI CellROX
T1
T2
T2
Neutrophils
MDSC

Figure EV3. Transiently emerging myeloid-derived suppressor cells.

(**A**) Flow cytometric gating strategy for immune phenotyping of $CD14^-HLA^-DR$-$CD11b^+CD15^+$ MDSC derived from patients during the course of major surgery. Representative scatter plots from a postoperative (T2) sample, as depicted in Fig. 3C. (**B**) Representative May–Grünwald–Giemsa staining of PBMC after major surgery. Transiently present cells depicting a granulocytic morphology (arrows). (**C**) Representative May–Grünwald–Giemsa staining of isolated MDSC, extracted from PBMC after major surgery using CD15 microbeads. (**D**) Flow cytometric analysis and immune phenotyping of PBMC from patients with minor surgery with gating strategy for detection of $CD14^-HLA^-DR$-$CD11b^+CD15^+$ MDSC. (**E**) Quantification of $CD14^-HLA^-DR$-$CD11b^+CD15^+$ MDSC as % of PBMC, derived from patients pre (T1) and post (T2) minor surgery, $n = 10$. (**F**) Heat map depicting quantity of all significantly differentially expressed transcripts of T2 MDSC vs T2 Granulocytes ($p < 0.0001$). Each pair of MDSC/granulocytes derived from the same patient. Red color indicates an upregulation, and downregulated genes are indicated by blue color. (**G**) Volcano plots visualizing differential gene expression T2 MDSC/ T2 granulocytes. Log2 fold-changes (x-axis) and −log10 adjusted *p* values (y-axis) are shown for each gene. The dashed horizontal line depicts the adj. *p* value threshold of $p < 0.0001$, significantly regulated genes in red (upregulated in MDSC) and green (downregulated in MDSC). (**H**) Gene Ontology (GO) analysis of differentially expressed pathways in T2 MDSC/ T2 Granulocytes. The gene counts are expressed by the numbers within the bubbles. (**I**) Flow cytometric analysis and quantification of intracellular ROS as indicated by CellROX green staining in sorted cell populations, time points as stated, $n = 6$ individual patients. Data were represented as mean ± SEM with dots representing individual values. *P* values as indicated, paired *t*-test.

 © The Author(s)

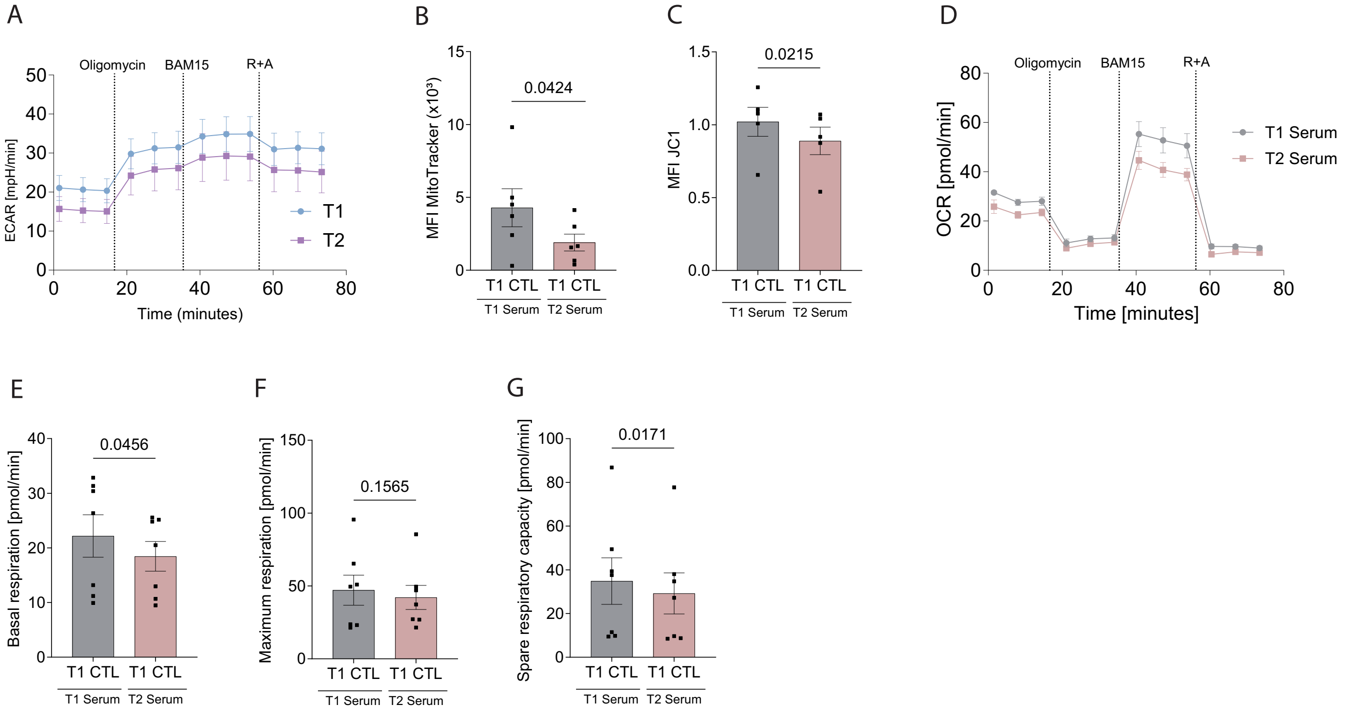

Figure EV4. T1 CTL cultivated with T2 MDSC or T2 serum mimic phenotype of T2 CTL mitochondrial damage.

(**A**) Pooled seahorse ECAR plot, mean ± SEM from five individual patients. (**B–G**) CTL of patients before major surgery (T1) co-cultivated with 20% serum from patients before (T1) or after (T2) major surgery. (**B**) FCA of MitoTracker green staining and quantification, $n = 6$ individual patients. (**C**) FCA of JC1 staining and quantification of red/green fluorescence ratio, $n = 5$ individual patients. (**D**) Representative seahorse OCR plot. (**E–G**) Seahorse quantification of (**E**) basal OCR, (**F**) maximal OCR, and (**G**) spare respiratory capacity in CTL, $n = 7$ from three individual patients. Data were represented as mean ± SEM with dots representing individual values. *P* values as indicated, paired *t*-test or Wilcoxon matched-pairs signed-rank test, as appropriate.

© The Author(s)

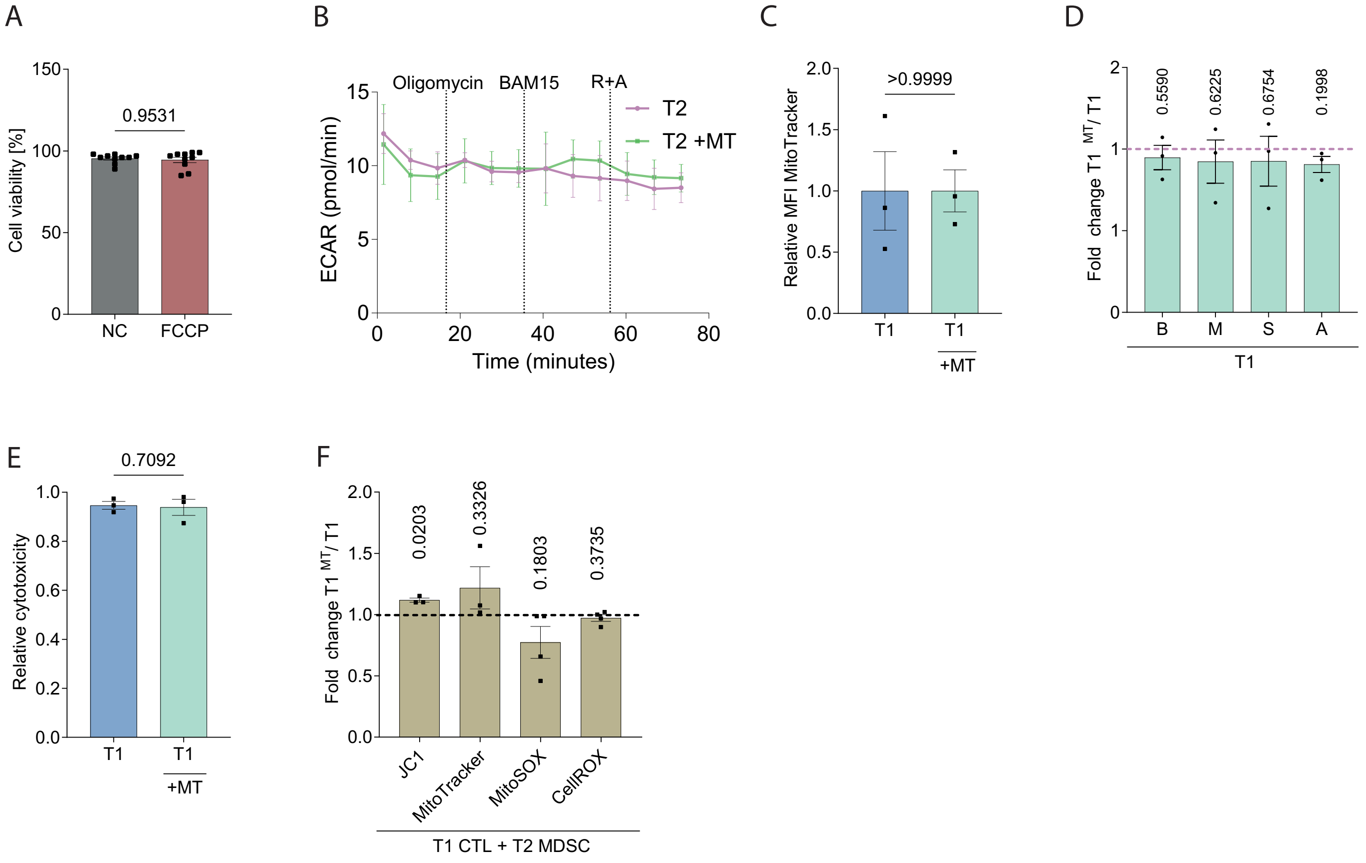

Figure EV5. No impact of FCCP on cell viability and no impact of mitochondrial antioxidative treatment using MitoTEMPO (MT) on T1 CTL immunometabolism.

(**A**) Cell viability of CTL treated with low-dose FCCP, $n = 10$, p value as indicated. (**B**) Pooled seahorse ECAR plot, mean ± SEM from six individual patients. (**C**) FCA of MitoTracker green and quantification in CTL, $n = 3$ individual patients. (**D**) Fold change of basal OCR (*B*), maximum OCR (*M*), spare respiratory capacity (*S*), and ATP production (*A*), $n = 3$ individual patients. (**E**) Quantification of relative CTL cytotoxicity using a flow cytometric killing assay, $n = 3$ individual patients. (**F**) Fold change of mitochondrial parameters as indicated comparing MT treated T1 CTL co-cultivated with MDSC versus untreated T1 CTL co-cultivated with MDSC, $n = 4/3/3/4$. Data were represented as mean ± SEM. *P* values as indicated, one-sample *t*-test (**D**) paired *t*-test or Wilcoxon matched-pairs signed-rank test, as appropriate.

 © The Author(s)
